# A cross-sectional study: correlation of forehead morphology and dentoskeletal malocclusion in Chinese people

**DOI:** 10.1186/s12903-023-03795-1

**Published:** 2024-01-08

**Authors:** Xiuyun Zheng, Siqi Ding, Qin Mei, Chuan Wu, Qunyan Zhang, Chunfeng Fu, Quancheng Han, Shiyu Jin, Ruiqi Yu, Muchen Yu, Zixian Ye, Jun Shen, Jianguang Xu, Xiaoyu Liu, Tingting Wu

**Affiliations:** Department of Orthodontics, College & Hospital of Stomatology, Anhui Medical University, Key Lab. of Oral Diseases Research of Anhui Province, 69 Meishan Road, Shushan District, Hefei, 230032 China

**Keywords:** Forehead protrusion, Lateral profile, Frontal sinus, Aesthetics, Retrospective study

## Abstract

**Background:**

The lateral profile is an important indicator of facial attractiveness. This study explored the general characteristics of the forehead profile and protrusion, and their relationship with related factors in structure and development.

**Methods:**

Four hundred fourteen Chinese participants in the Yangtze River Delta region were involved. Including 206 males (17.15 ± 7.68 years old) and 208 females (18.35 ± 8.06 years old); 94 children (8.54 ± 2.21 years old, ranging from 4 to 12 years old), 166 adolescents (14.83 ± 1.50 years old, ranging from 13 to 17 years old), and 154 adults (25.52 ± 4.89, 18 years or older). The frontal section of the forehead was used to explore its shape. The straight distance between the vertical line of the FH plane through the nasal root point and its parallel line, which is tangential to the forehead, indicates the forehead prominence. Frontal sinus width was measured using the method described by Mahmood.

**Results:**

The general shape of the forehead was straight and slightly bulged near the eyebrow arch in males but rounder in females. The average forehead protrusion in males was higher than that in females in adults. Significant differences in forehead protrusion between the dentoskeletal classifications and growth phases were notable. Frontal protrusion significantly correlated with frontal sinus depth, especially in males, adults, Class I, and those whose convex points were located in the lower section of the forehead.

**Conclusions:**

Age, race, and sex affect the forehead protrusion and frontal sinus width. Forehead protrusion may be an indicator of dentoskeletal deformities in the early stage. And dentoskeletal deformities may impair the correlation between the frontal sinuses and forehead protrusion during development.

**Trial registration:**

This retrospective, cross-sectional study was reviewed and approved by the Research Ethical Committee (T2020008), and registered at ClinicalTrial.gov with an identified number (ChiCTR2100041913).

## Background

Orthodontics and orthognathic surgery are based on aesthetics. Greater emphasis has been placed on the attractiveness of appearance in recent years [1, 2]. The desire to appear more attractive is the primary motivation of patients undergoing treatment [3, 4]. Many studies have demonstrated the positive effects of orthodontic and orthognathic treatment on the lives of patients with dentofacial deformity [5–7]. Today, orthodontic treatment aims to pay more attention to facial proportions and the impact of dentition on appearance, forming a soft tissue-oriented treatment [8]. The soft tissue paradigm states that orthodontic treatments are determined by the overall structure of the face, rather than by the local structure, so factors associated with the attractiveness of the soft tissue profile need to be assessed [9].

The lateral profile is an important indicator of facial attractiveness. The esthetics of the profile have received increasing attention, studies have been conducted to evaluate the effect of local structures on soft tissue profiles, such as the position and protrusion of lips, which are related to the arrangement of teeth [10, 11].

Whereas most research has concentrated on the lower third of the face, the upper third, including the brow, forehead, and temple, provides an important contribution to the overall facial esthetic [12]. The upper face is a key transmitter of nonverbal messaging and imparted emotion system [13, 14]. A survey on the esthetic favor showed that the upper 1/3 of the face was the most important region [15]. One research has reported that different favors exist in the same silhouettes of the face with or without the forehead, suggesting that the shape of the forehead affects the aesthetics in the overall lateral appearance [16]. Another study found that the aesthetic preference for the lateral view of the mandibular relationships changed when adjusting the protrusion of the forehead [17]. All these studies suggested that forehead plays an integral role in facial aesthetics.

Despite the important status of forehead, relevant studies are rare. Powell’s aesthetic triangulation started with the forehead to analyze the major aesthetic elements of the face [18]. Adults with normal occlusion met the esthetic requirements of the principle of Andrews’ six elements of orofacial harmony which involves the forehead [19, 20]. Therefore, frontal esthetic factor research based on the anterior-posterior position of the maxillofacial bone is worth exploring. Moreover, Kocandrlova [21] and Brons [22] indicated that the forehead shape might differ among sexes and growth phases in preschool children and infants. However, the related factors affecting frontal morphology remain unclarified.

The frontal sinus is located in the posterior portion of the superciliary ridge between the endocranial and ectocranial tables of the frontal bone and is an air-filled cavity located on the frontal bone of the skull [23]. Frontal sinus growth is synchronous with craniofacial growth, and its enlargement is based on the expanding braincase [24]. Therefore, clarifying the relationship between the forehead and frontal sinuses is particularly important.

In sum, our hypothesis for this study was that there might be a relationship between the forehead protrusion and the lateral profile of participants. So, the present study was conducted to ascertain the pattern of growth and anatomical structure with regard to the profile and protrusion of the forehead, and further explore application in clinical practice.

## Methods

The study participants were selected from Chinese people in the Yangtze River Delta region of east China. Samples were obtained from the orthodontic department between 2016 and 2023, and complete medical history data were collected. Subjects with good-quality standardized pre-treatment lateral cephalograms and photographs were included in this study. The exclusion criteria were as follows: 1) cranial deformities due to development, tumors, trauma, or inflammation; 2) history of orthodontic treatment, facial plastic surgery, or orthognathic surgery before this treatment; and 3) previous treatments interfering with growth.

The experimental materials included a 90-degree profile photograph and a lateral cephalometric radiograph. All the patients underwent consistent, standardized procedures. Lateral facial photographs were obtained with a digital camera (EOS 90D; Canon, Tokyo, Japan) in an illuminated studio environment against a white background. The camera was placed at the same height as the head and 2 m away from the participants; the lens was parallel to the individual’s sagittal plane. The screening criteria were as follows: median sagittal plane parallel to the background screen plane and Frankfurt plane parallel to the ground plane; facial muscles relaxed naturally; hair brushed behind the ears, fully exposing the forehead and auricle. The teeth were placed in a resting position with the lens level to the ear. Photographs were analyzed, selected, and standardized using Photoshop (Adobe Systems Inc., San Jose, California, USA).

All lateral cephalometric radiographs were standardized using an Orthoralix R9200 (Gendex-KaVo, Milan, Italy). The subject was upright, with the head muscles naturally relaxed, eyes looking straight ahead, teeth naturally clenched, lips naturally closed, and mechanical earplugs on both sides well aligned. Cephalograms were obtained with rigid head fixation and a 165 cm film-to-tube distance.

The sample size was calculated to be 96, with a confidence level of this study set as 95%, an alpha of 0.05, and an accuracy level of 10%. To increase the power of the study, we included the maximum number of available participants. With informed consent, the final samples were 414 after screening according to the above criteria. All samples were classified according to age, sex, dentoskeletal classification, and the position of the most prominent point of the forehead. There were 206 males (17.15 ± 7.68 years old) and 208 females(18.35 ± 8.06 years old) involved. When classified by age, the mean age of the children samples was 8.54 ± 2.21 years old (ranging from 4 to 12 years old), for adolescents was 14.83 ± 1.50 years old (ranging from 13 to 17 years old), and for adults was 25.52 ± 4.89 (18 years or older). When classified by dentoskeletal classification, an angle (∠ANB) between 0 and 4° is defined as Class I, an angle (∠ANB) greater than 5° is defined as Class II, and an angle (∠ANB) less than 0° is defined as Class III. To investigate the relationship between frontal protrusion and frontal sinus width, we vertically divided the forehead into upper, middle, and lower parts. Based on the position of the most protruding point of the forehead, we divided all samples into three categories: upper 1/3 convex, middle 1/3 convex, and lower 1/3 convex.

### Forehead shape

**Fig. 1:**
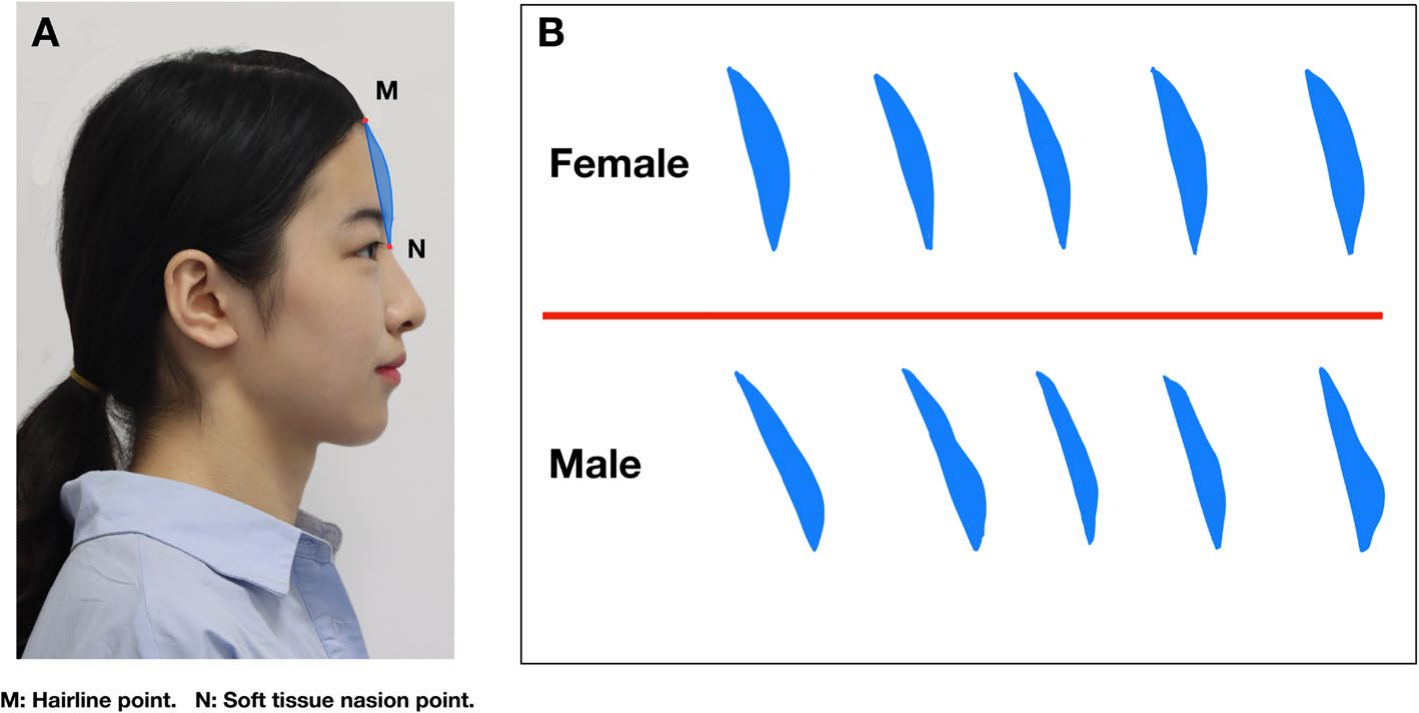
The shape of the forehead: A, drawing method of the frontal section of the forehead; B, forehead shape. M, hairline point; N, soft tissue nasion point

We studied the shape characteristics of the outer forehead contour in different subjects by exploring the shape of the frontal section of the forehead. Our adopted method focused on the frontal section between the natural hairline, soft tissue nasion, and forehead contour. The general shape of the forehead was traced following the photograph (Fig. 1).

### Measurement of forehead protrusion value

Through the observation of 414 photographs, most of the prominent parts of the forehead were concentrated in the middle, and lower sections of the forehead, and this section can be seen on lateral radiographs. Therefore, we can measure frontal protrusion in the middle and lower sections of the forehead using lateral radiographs, and the frontal protrusion value can be defined by the fixed location of the middle and lower sections of the forehead in the FH plane direction. The section of the forehead where the frontal bone joins the nasal bone is called the nasofrontal suture, where the nose’s root is located and is the most concave point in the entire forehead. The contrast of the lateral radiographs was adjusted to show soft tissue contours. The vertical Line L1 of the FH plane was made through the nasal root point, and the parallel line when the line was tangential to the forehead of the soft tissue was defined as L2. The tangent point is defined as “the bulging point.”

**Fig. 2:**
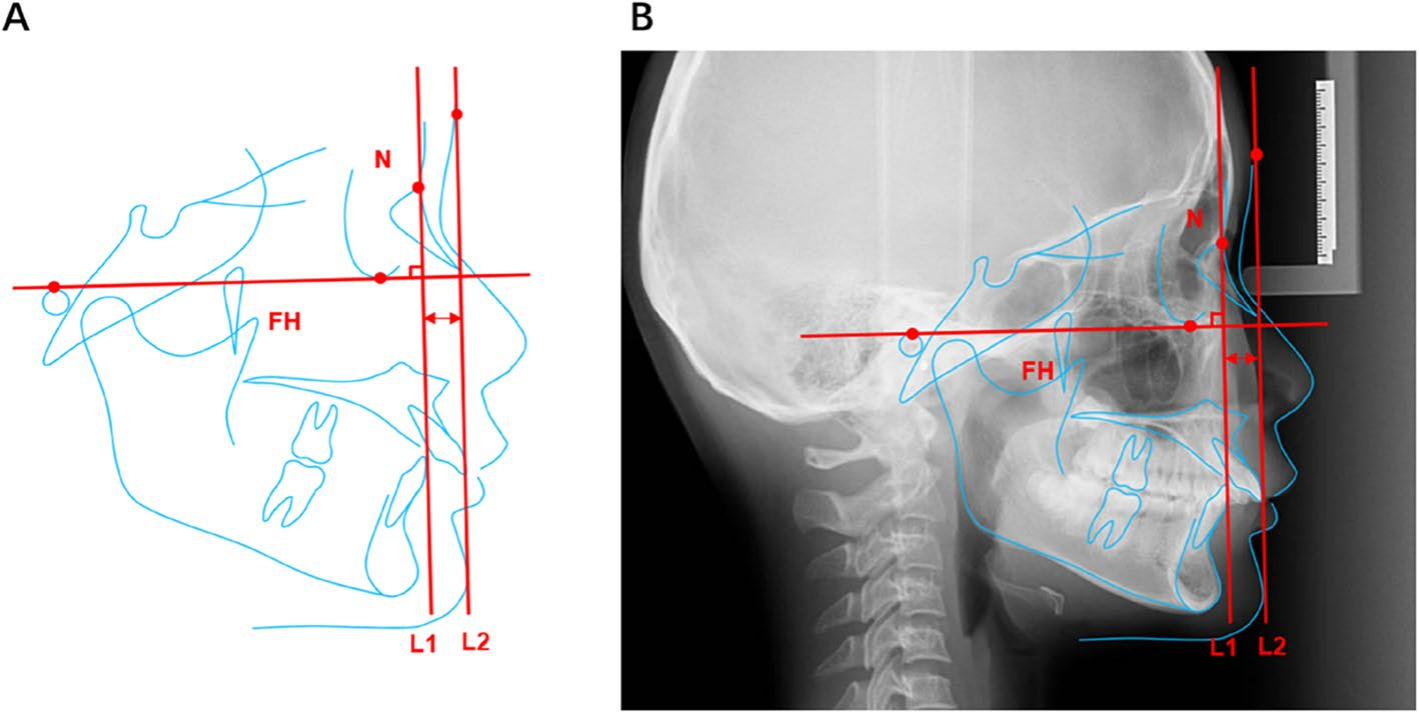
Measurement method of the forehead protrusion. **a**, schematic view; **b**, lateral cephalometric radiograph view. FH, the Frankfurt horizontal plane; N, nasion; L1, the vertical Line of the FH plane through the nasion; L2, the parallel line of L1 tangent to the soft tissue forehead. The straight distance between the parallel Lines L1 and L2 indicates forehead prominence. (The distance indicated by the red arrow)

The straight distance between the parallel Lines L1 and L2 indicates forehead prominence (Fig. 2). According to the above overlapping and definition method, the value of forehead protrusion in each profile photograph was measured, and the actual value of forehead protrusion was obtained by conversion, using the positioning scale of the lateral cranial X-rays as the standard. The FH plane was determined by three orthodontic specialists with > 10 years of experience. Line drawing was performed independently by a researcher who had undergone rigorous training, the distance between the two parallel lines for each subject was measured twice with ImageJ by the two researchers, using the average of the four measurements as the final value for forehead protrusion. Interclass correlation coefficient (ICC) was calculated to assess the agreement of the measurement between two researchers as well as the consistency of multiple measurements by the same researcher. It is recommended that the ICC should be greater than 0.80, with 0.61 to 0.80 being medium, 0.41 to 0.60 being fair, 0.11 to 0.40 being low, and 0.1 or less being no consistency.

### Frontal sinus width

**Fig. 3:**
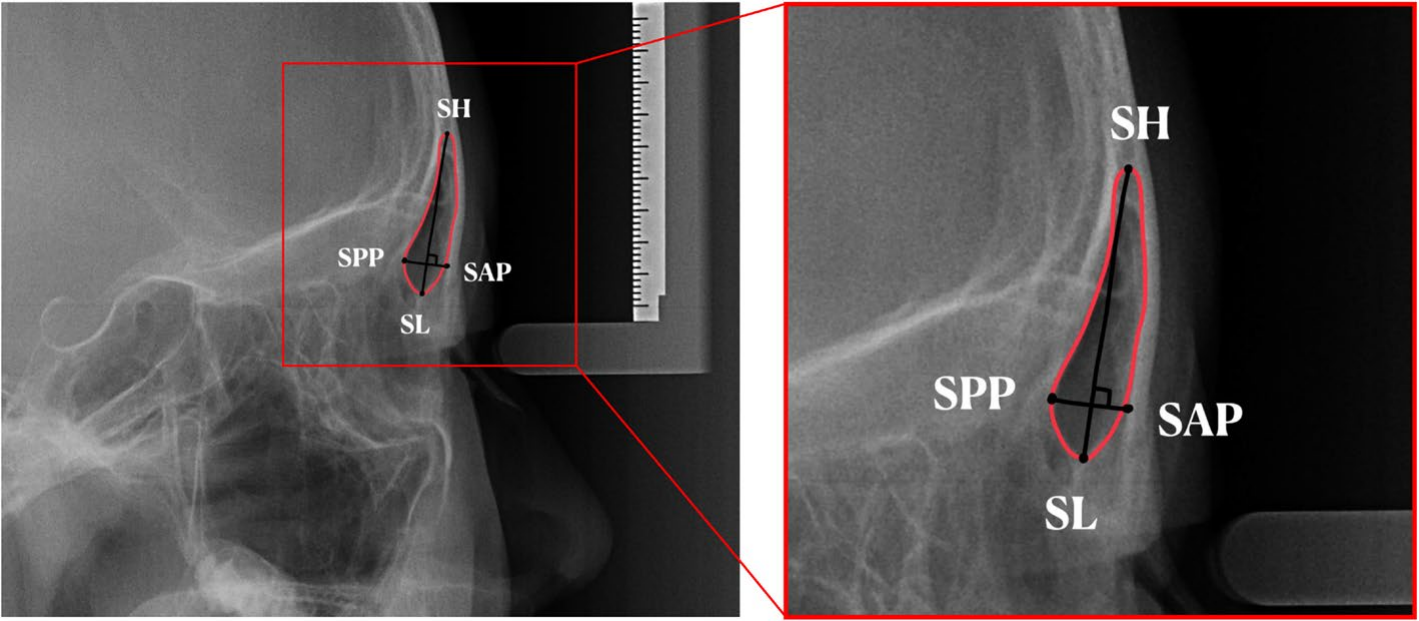
The contour of frontal sinus and measurement method of frontal sinus width. SH, the highest point on the frontal sinus; SL, the lowest point on the frontal sinus; SH-SL, maximum frontal sinus height; SPP, the posterior point on the frontal sinus; SAP, the anterior point on the frontal sinus; SPP-SAP, joining the SPP and SAP, denoting the maximum frontal sinus width perpendicular to the SH-SL line

Most frontal sinus structures can be identified using lateral cephalometric radiographs. We traced the contours of the frontal sinus can be detected in lateral cephalometric radiographs. The frontal sinus widths of the subjects in this study were plotted by referring to the method used in Mahmood’s literature [25] (Fig. 3). Frontal sinus morphology was assessed on a lateral cephalogram using the method described by Mahmood et al.. SH, the highest point on the frontal sinus; SL, the lowest point on the frontal sinus; SH-SL, maximum frontal sinus height; SPP, the posterior point on the frontal sinus; SAP, the anterior point on the frontal sinus; SPP-SAP, joining the SPP and SAP, denoting the maximum frontal sinus width perpendicular to the SH-SL line. The same highly trained researcher performed this task, and the final value of the frontal sinus width was obtained by averaging multiple measurements.

### Statistical analysis

Statistical analyses were performed using SPSS (version 26; IBM, Armonk, NY, USA) and GraphPad Prism software (version 9.5.0; GraphPad Software, San Diego, California, USA). For each category group, descriptive statistics were documented (i.e., mean and SD were calculated for sample composition and forehead protrusion values in different categories). The normality of the data was determined using the Kolmogorov-Smirnov test. A rank-sum test was used for samples that were not normally distributed. The equality of variances was checked using Levene’s test. Statistical analyses of forehead protrusion and frontal sinus width and their relationship with age, sex, and dentoskeletal classification were performed using the independent samples t-test and one-way ANOVA test.

Pearson’s two-variable analysis was used to analyze the correlation between frontal sinus width and forehead protrusion. To relate the consistency and reliability of the two measurements, an interclass correlation coefficient (ICC) was calculated based on a two-way mixed-effects model that was consistent within and between the measurement value groups. The results were evaluated using 95% confidence intervals. Statistical significance was set at *P* < 0.05.

## Results

### The patterns of forehead shapes

**Fig. 4:**
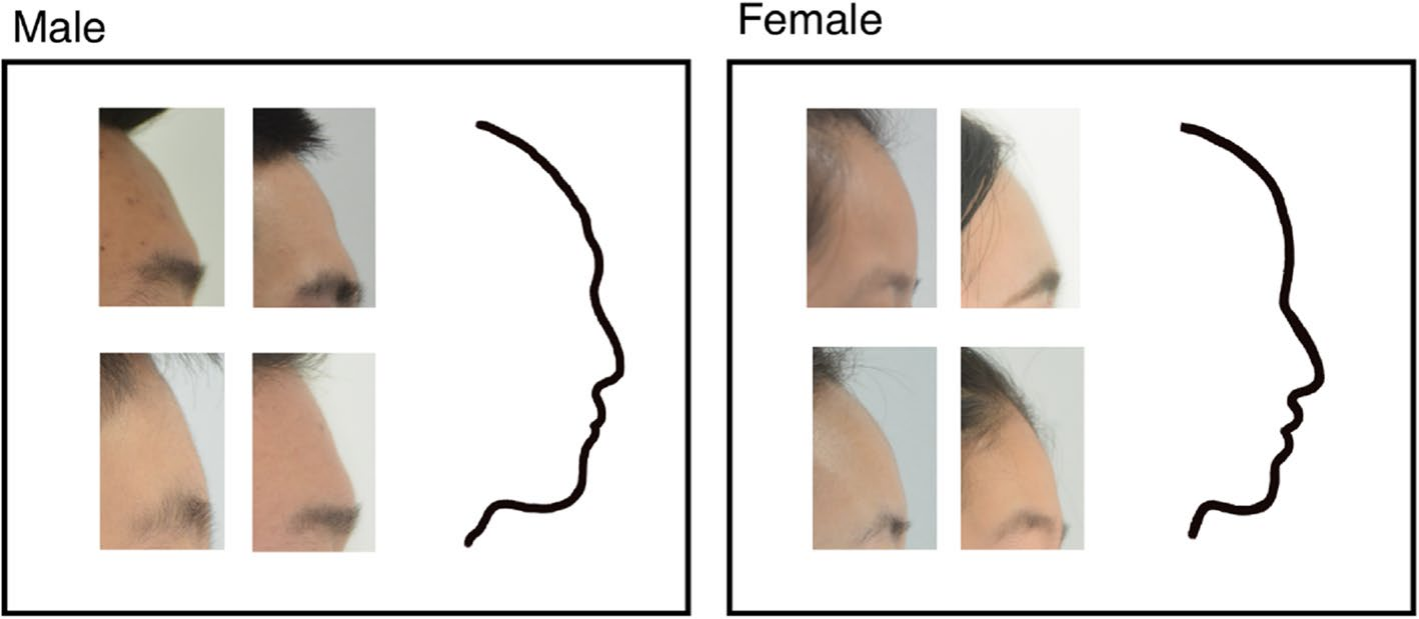
The contour of the forehead

Drawing the shape of the forehead showed a significant difference in the general shape of the forehead between the sexes (Fig. 4). The forehead is similar to the arc of a circle, and women’s foreheads are more rounded, with the most protruding point in the middle; in men, the middle and upper part is inclined, and “the bulging point” tends to be lower. Females have a rounded forehead with a slight bulge in the middle; male foreheads are flat and relatively high near the eyebrow arch; sometimes, the male forehead has two bimodal bumps, and the lower bulge is higher than the upper one. Following this sex-specific forehead trend, we sketched the contours of males and females.

### The forehead protrusion value

A total of 414 participants consisting of 210 dentoskeletal class I, 128 class II, and 76 class III patients were recruited for this study. Two hundred six men and 208 women were involved in this study. The percentage of teenagers is similar to that of adults, with 40% in teenagers and 37.3% in adults, the rest is shared by children with a percentage of 22.7%. The ICC of the two researchers’ measurements was 0.959 (*P* < 0.000), and the ICC for each individual was 0.998 (*P* < 0.000) and 0.988 (*P* < 0.000), respectively, indicating good agreement between the two researchers’ measurements.

**Fig. 5:**
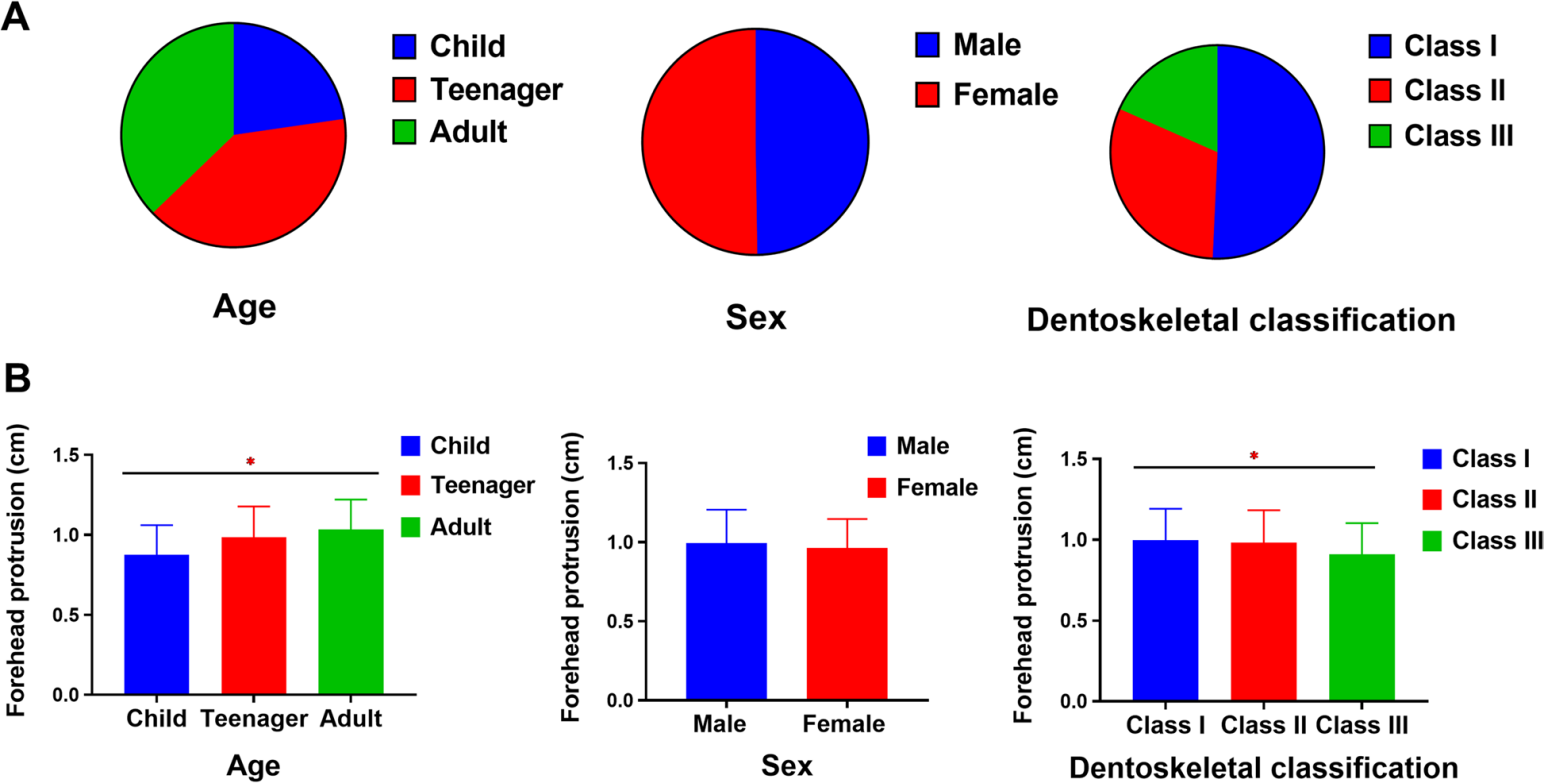
The average frontal protrusion: A, the consists of all samples; B, the average frontal protrusion in different categories of all samples. *: *P* < 0.05 between groups. Error bars represent means ± standard deviations

From this study, the average frontal protrusion in each group was statistically analyzed according to sex, age, and dentoskeletal category. The results showed statistically significant differences in the mean values between age and dentoskeletal categories, but there was no significant difference in the sex groups (Fig. 5).

Specifically, it can be concluded that there was no statistically significant difference between the sexes in forehead protrusion, the average value of females was 0.962 ± 0.184 cm, and the average value of males was 0.995 ± 0.210 cm. We continued to explore the influence of related factors such as age and dentoskeletal classifications based on sex. The results showed that the frontal protrusion of males was higher than that of females only in adults (*P* < 0.001), suggesting that males and females are not synchronized in the development of the forehead. However, no statistical significance in dentoskeletal categories (Table 1a).

**Table 1:** Means, SDs of the value of forehead protrusion, and mean differences between sexes (a), between growth phases (b), and that for dentoskeletal classifications (c)

**(a)**	Number			Mean ± SD of forehead protrusion (cm)			*P* value
Index	Male	Female		Male		Female	
All samples	206	208		0.995 ± 0.210		0.962 ± 0.184	0.096
Age							
Child	52	42		0.854 ± 0.168		0.902 ± 0.204	0.216
Teenager	91	75		0.994 ± 0.192		0.976 ± 0.192	0.548
Adult	63	91		1.111 ± 0.196		0.979 ± 0.161	< 0.001**
Dentoskeletal Classification							
Class I	103	107		1.022 ± 0.219		0.978 ± 0.219	0.094
Class II	66	62		1.000 ± 0.210		0.967 ± 0.189	0.356
Class III	37	39		0.907 ± 0.161		0.912 ± 0.222	0.920
**(b)**	Number			Mean ± SD of forehead protrusion (cm)			P value
Index	Child	Teenager	Adult	Child	Teenager	Adult	
All samples	94	166	154	0.876 ± 0.185	0.986 ± 0.192	1.033 ± 0.188	0.001*
Sex							
Male	52	91	63	0.854 ± 0.168	0.994 ± 0.192	1.111 ± 0.196	< 0.001**
Female	42	75	91	0.902 ± 0.204	0.976 ± 0.192	0.979 ± 0.161	0.058
Dentoskeletal Classification							
Class I	29	94	87	0.873 ± 0.182	0.986 ± 0.192	1.057 ± 0.176	< 0.001**
Class II	30	50	48	0.808 ± 0.133	1.020 ± 0.195	1.055 ± 0.177	< 0.001**
Class III	35	22	19	0.936 ± 0.209	0.905 ± 0.169	0.867 ± 0.192	0.455
**(c)**	Number			Mean ± SD of forehead protrusion (cm)			P value
Index	Class I	Class II	Class III	Class I	Class II	Class III	
All samples	210	128	76	0.999 ± 0.193	0.984 ± 0.199	0.910 ± 0.193	0.003*
Sex							
Male	103	66	37	1.022 ± 0.219	1.000 ± 0.210	0.907 ± 0.161	0.016*
Female	107	62	39	0.978 ± 0.162	0.967 ± 0.189	0.912 ± 0.184	0.154
Age							
Child	29	30	35	0.873 ± 0.182	0.808 ± 0.133	0.936 ± 0.209	0.020*
Teenager	94	50	22	0.986 ± 0.192	1.020 ± 0.195	0.905 ± 0.169	0.063
Adult	87	48	19	1.057 ± 0.176	1.055 ± 0.177	0.867 ± 0.191	<0.001**

*SD* standard deviation

*Statistical significance (*P* < 0.05) as determined by the Independent-Samples T-Test and analysis of variance

**Highly statistically significant (*P* < 0.001)

In all samples, the average value of forehead protrusion detected a statistically significant interaction between ages (*P* = 0.001), and the value was 0.876 ± 0.185 cm in children, 0.986 ± 0.192 cm in teenagers, and 1.033 ± 0.188 cm in adults. We categorized the subgroups according to sex and dentoskeletal categories based on age (Table 1b). Significant differences between growth phases were notable in the males (*P* < 0.001), suggesting that the forehead constantly develops at all ages in males, whereas in females it develops significantly during puberty. Differences in forehead protrusion between growth phases were significant in the dentoskeletal Class I (*P* < 0.001) and the Class II (*P* < 0.001) categories, this suggests that there may be some relationship in terms of the cranial and maxillofacial development.

Among all the participants, the average value of forehead protrusion detected a statistically significant interaction between the dentoskeletal categories (*P* < 0.05), and the value was 0.999 ± 0.193 cm in Class I, 0.984 ± 0.199 cm in Class II and 0.910 ± 0.193 cm in Class III. Based on the different dentoskeletal categories, the average differences between sexes and age groups were studied. Significant differences between dentoskeletal categories were notable in the males (*P* < 0.05), children (*P* < 0.05), and adults (*P* < 0.001) (Table 1c). It suggested that forehead protrusion of Class III in children was more pronounced compared to that of Classes I and II, whereas the average forehead protrusion of dentoskeletal Class III was the smallest in adults. To some extent, there may be some regulatory mechanism affecting mandibular development after the process of forming a pronounced forehead in children. The overdeveloped mandible, in turn, may inhibit or retard the development of the frontal bone and maxilla mediated by some mechanism during adolescence. It ultimately leads to dentoskeletal Class III malocclusion, and forms a profile of a protruding mandible with a flat forehead in adulthood. It means children with a rounder forehead are more likely to develop a dentoskeletal Class III malocclusion in the future. Meanwhile, individuals with smaller forehead protrusions as children may have an increased risk of developing dentoskeletal Class II malocclusions as adults. Hence, the forehead protrusion may be an indicator of dentoskeletal malocclusion in the early stage.

For all statistically significant classifications, we further analyzed the relationship within groups (Table 2). Between growth phases, no difference between teenagers and adults in the dentoskeletal Class II and females, whereas significant differences between growth phases were noted between all samples, males, and the dentoskeletal Class I, whereas no correlation was found in Class III and age-related development.

**Table 2:** Pairwise comparison among growth phases (a) and dentoskeletal classifications (b) of forehead protrusion

**(a)**	*P*		
Index	Child-Teenager	Child-Adult	Teenager-Adult
All samples	<0.001**	<0.001**	0.025*
Sex			
Male	<0.001**	<0.001**	< 0.001**
Female	0.037*	0.025*	0.908
Dentoskeletal Classification			
Class I	0.004*	<0.001**	0.010*
Class II	<0.001**	<0.001**	0.325
Class III	0.563	0.213	0.526
**(b)**	P		
Index	Class I-Class II	Class I-Class III	Class II-Class III
All samples	0.465	<0.001**	0.009*
Sex			
Male	0.483	0.004*	0.031*
Female	0.053	0.213	0.526
Age			
Child	0.170	0.165	0.005*
Teenager	0.299	0.075	0.019*
Adult	0.953	<0.001**	<0.001**

*SD* standard deviation

*Statistical significance (*P* < 0.05) as determined by the ANOVA test

**Highly statistically significant (*P* < 0.001)

### Relationship between frontal sinus width and forehead protrusion

**Fig. 6:**
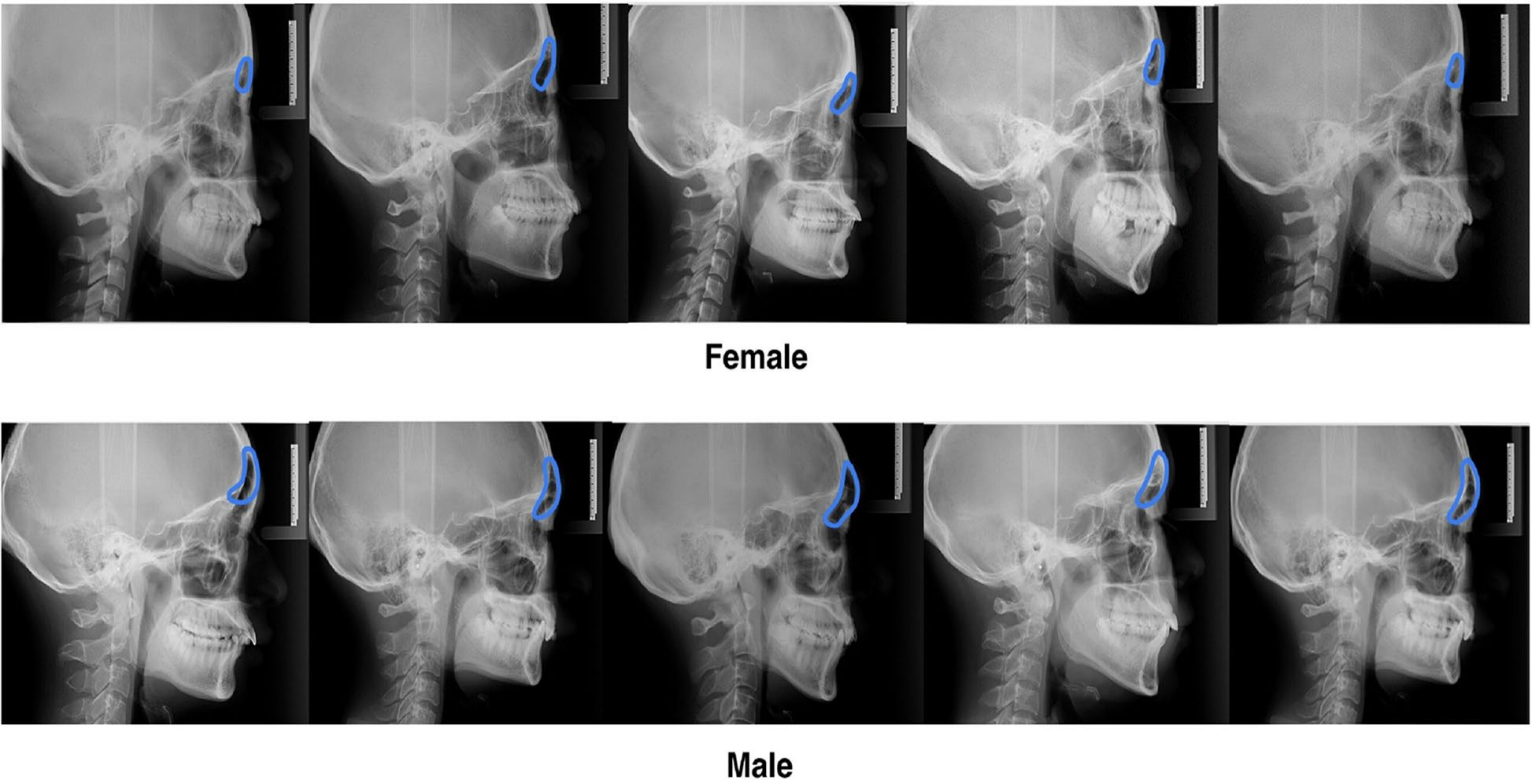
The shape of the forehead sinus

From the previous analysis, we found that the frontal protrusion of males was greater than that of females in adults, and drawings of the shape of the frontal sinus showed that the frontal sinus of males was larger than that of females (Fig. 6). Hence, the relationship between the frontal sinuses and the forehead is worth considering.

All 405 participants were those who could trace the shape of the frontal sinus, and excluded nine individuals who did not meet the requirements. The maximum anterior-posterior diameter of the frontal sinuses was measured on lateral cephalometric radiographs. To investigate the relationship between forehead protrusion and frontal sinus depth, we divided the forehead into upper, middle, and lower parts vertically and divided all samples into three types according to the position of the most protruding point of the forehead. Of the 206 males, 143 had a protruding lower forehead section. Of the 63 adult males, 58 had a convex shape in the lower 1/3 section of the forehead, and we found that the most protruding point in males was mostly located in the lower section of the forehead.

Across all samples, the average frontal sinus depth was 0.840 ± 0.269 cm. When grouped by sex, the width of the frontal sinus was significantly greater in males (0.936 ± 0.291 cm) than in females (0.744 ± 0.204 cm, *P* < 0.001). When grouped by age, the frontal sinus depth of adults is the largest (0.880 ± 0.269 cm), and the frontal sinus depth of children is significantly smaller than that of adults and adolescents, indicating that there may be an obvious development of the frontal sinus during adolescence. The frontal sinus width of the participants with the most protruding point in the lower part (0.917 ± 0.293 cm) was greater than that of the participants with the most protruding point in the upper (0.757 ± 0.248 cm, *P* < 0.05) and middle parts (0.764 ± 0.217 cm, *P* < 0.001). There was no statistically significant difference in bone classification, indicating that the frontal sinus depth had little relationship with dentoskeletal malocclusion (Table 3).

**Table 3:** Frontal sinus width of all samples and pairwise comparison among subgroups

Index	Sex		Dentoskeletal classification			Age			The most convex part of the forehead		
	Male	Female	Class I	Class II	Class III	Child	Teenager	Adult	Upper1/3	Middle1/3	Lower1/3
N	204	201	204	127	74	93	160	152	14	187	204
Mean ± SD	0.936 ± 0.291	0.744 ± 0.204	0.849 ± 0.287	0.820 ± 0.277	0.855 ± 0.277	0.766 ± 0.230	0.848 ± 0.250	0.880 ± 0.269	0.757 ± 0.248	0.764 ± 0.217	0.917 ± 0.293
*P*	<0.001**		0.563			0.005*			<0.001**		
Pairwise comparison	Male-Female		I-II	II-III	I-III	C-T	T-A	A-C	U-M	M-L	U-L
	<0.001**		0.340	0.377	0.876	0.019*	0.282	0.001*	0.084	0.000**	0.017*

The N in all categories refers to cases in which the maximum anterior-posterior diameter of the frontal sinus can be traced and measured in the lateral cephalometric radiograph I, II, III; A, T, C; U, M, L: Means Class I, Class II, Class III; Adult, Teenager, Child; Upper, Middle, Lower

*SD* standard deviation

* Statistical significance (*P* < 0.05) as determined by the Kruskal-Wallis test and Independent-Samples T-Test

** Highly statistically significant (*P* < 0.001)

Data analysis of all samples showed that frontal protrusion was significantly correlated with frontal sinus depth (*P* < 0.05). A significant association was found between the frontal sinus width and forehead protrusion in males (*P* < 0.05) and in adults grouped by age (*P* < 0.001), indicating that frontal sinus and cranial bones may be more stable in adulthood. There was a correlation between frontal sinus width and frontal protrusion in Class I based on the dentoskeletal classification (*P* < 0.05). When considering different forehead shapes, the samples where the most protruding point was located in the lower sections followed the same pattern (*P* < 0.05) (Table 4), probably because the frontal sinus was also located closer to the lower part of the forehead, and the frontal sinus more influenced the forehead.

**Table 4:** Relationship between frontal sinus width and forehead protrusion of all samples

Index	Number	Mean ± SD (cm)		*P*
		frontal sinus width	forehead protrusion	
All samples	405	0.841 ± 0.269	0.975 ± 0.192	0.002*
Sex				
Male	204	0.937 ± 0.291	0.995 ± 0.211	0.013*
Female	201	0.743 ± 0.204	0.956 ± 0.170	0.587
Age				
Adult	152	0.880 ± 0.301	1.033 ± 0.189	<0.001**
Teenager	160	0.848 ± 0.250	0.983 ± 0.181	0.660
Child	93	0.766 ± 0.230	0.868 ± 0.172	0.968
Dentoskeletal classification				
Class I	204	0.849 ± 0.287	1.002 ± 0.194	0.009*
Class II	127	0.820 ± 0.233	0.977 ± 0.185	0.118
Class III	74	0.855 ± 0.277	0.899 ± 0.180	0.320
Forehead’s most convex part				
In the upper 1/3	14	0.757 ± 0.248	1.019 ± 0.262	0.930
In the middle 1/3	187	0.764 ± 0.217	0.974 ± 0.173	0.179
In the lower 1/3	204	0.917 ± 0.293	0.974 ± 0.204	0.002*

The Number in all categories refers to cases in which the maximum anterior-posterior diameter of the frontal sinus can be traced and measured in the lateral cephalometric radiograph

*Statistical significance (*P* < 0.05) as determined by Pearson’s two-variable analysis

**Highly statistically significant (*P* < 0.001)

Analysis of males and adults showed that only the frontal sinus width in Class I and the lowest protruding forehead position were related to frontal protrusion, of Class I, males, adults, and the lowest protruding forehead position exhibited correlations. We analyzed individuals with the lowest protruding forehead position, and the subgroups of males(*P* = 0.027), adults (*P* < 0.05), and Class I (*P* < 0.05) exhibited correlations, indicating that the development of the frontal sinuses and forehead protrusion may be related to the development of a disproportionate anterior-posterior maxillofacial bone (Table 5).

**Table 5:** Relationship between frontal sinus width and forehead protrusion of males (a), adults (b), dentoskeletal classification I (c), and whose most convex position of the forehead in the lower 1/3 part (d)

Index	Number	Mean ± SD (cm)		*P*
		frontal sinus width	forehead protrusion	
**(a) Male**	204	0.937 ± 0.291	0.995 ± 0.211	0.013*
Age				
Child	52	0.825 ± 0.245	0.854 ± 0.168	0.655
Teenager	89	0.907 ± 0.254	0.995 ± 0.194	0.589
Adult	63	1.071 ± 0.326	1.111 ± 0.196	0.353
Dentoskeletal classification				
Class I	101	0.967 ± 0.300	1.024 ± 0.220	0.026*
Class II	66	0.876 ± 0.270	1.000 ± 0.210	0.159
Class III	37	0.963 ± 0.291	0.907 ± 0.161	0.841
**(b) Adult**	152	0.880 ± 0.301	1.033 ± 0.189	<0.001**
Sex				
Male	63	1.070 ± 0.326	1.111 ± 0.196	0.353
Female	89	0.977 ± 0.163	0.745 ± 0.191	0.318
Dentoskeletal classification				
Class I	85	0.886 ± 0.323	1.058 ± 0.178	<0.001*
Class II	48	0.870 ± 0.269	1.055 ± 0.177	0.157
Class III	19	0.877 ± 0.292	0.866 ± 0.191	0.545
**(c) Class I**	204	0.849 ± 0.287	1.002 ± 0.194	0.009*
Sex				
Male	101	0.967 ± 0.300	1.023 ± 0.220	0.026*
Female	103	0.734 ± 0.220	0.981 ± 0.163	0.828
Age				
Child	29	0.751 ± 0.283	0.873 ± 0.182	0.738
Teenager	90	0.845 ± 0.245	0.991 ± 0.192	0.457
Adult	85	0.886 ± 0.323	1.058 ± 0.178	<0.001**
**(d) In the lower 1/3**	204	0.917 ± 0.293	0.974 ± 0.204	0.002*
Gender				
Male	143	0.975 ± 0.306	1.006 ± 0.210	0.027*
Female	61	0.781 ± 0.203	0.899 ± 0.166	0.950
Age				
Child	39	0.837 ± 0.255	0.844 ± 0.158	0.635
Teenager	78	0.886 ± 0.264	0.954 ± 0.184	0.804
Adult	87	0.980 ± 0.321	1.050 ± 0.206	0.004*
Dentoskeletal classification				
Class I	100	0.949 ± 0.306	1.020 ± 0.205	0.009*
Class II	61	0.869 ± 0.271	0.982 ± 0.193	0.112
Class III	43	0.910 ± 0.285	0.856 ± 0.160	0.625

The Number in all categories refers to cases in which the maximum anterior-posterior diameter of the frontal sinus can be traced and measured in the lateral cephalometric radiograph

*Statistical significance (*P* < 0.05) as determined by Pearson’s two-variable analysis

**Highly statistically significant (*P* < 0.001)

## Discussion

The present study was conducted to determine the characteristics of the forehead by analyzing its profile and protrusion; exploring the influence of related factors such as age, sex, and dentoskeletal classification; and quantifying these possible differences.

### Forehead profile

With increased forehead protrusion in males during adolescence, males have a more significant protrusion than females in adulthood, and most males have a bulge at the lower part of the forehead. It indicated that the forehead morphology has a distinct sex difference. That is, males have a greater and more prominent supraorbital ridge that slopes backward toward the hairline, giving them a more angled and sharper upper face. These findings are similar to those of Jonathan M. Sykes [26] which found that the female forehead is slightly bent back at an angle of approximately 7°; in men, the angle of post-curvature is larger at approximately 10°. Research evidence combined with our investigation indicates that male foreheads may be more forward in the sagittal direction than female foreheads.

### Forehead protrusion

Relevant studies are rarely referred to the forehead in the aesthetic assessment. Holdaway’s [27] and Legan’s [28] soft tissue analyses are the most commonly used lateral soft tissue evaluation methods. These two methods only briefly mention the forehead in the evaluation of face shape, and the focus remains on the lower third of the face. Quantitative measurements of the forehead are not mentioned. McKinney [29] determined the forehead height, measured from the top of the eyebrow to the hairline (trichion), to be an average of 5 cm for women and 6 cm for men. However, considering the common occurrence of early receding hairlines in men and the ratio of brow height manipulation through makeup in women, the true value of such measurements is questioned [30]. Mahmood’s measurement [25] that we used has a better ability to be quantitatively analyzed with a high degree of measurement reproducibility. The more stabilized methods and materials waiting for exploration in the future study.

In the present study, participants with different dentoskeletal malocclusion classifications differ in forehead protrusions, which supports the existing study that aesthetic preference for mandible profile changes when adjusting forehead prominence [16, 17]. Moreover, Kocandrlova [21] has reported that the lateral lower part of forehead was more prominent in males than in females at every age group. However, our study demonstrated that sex differences in forehead protrusion are only manifested in adulthood. Ethnic influence with different developmental processes. May contribute to this discrepancy.

Furthermore, our present study suggested that forehead protrusion may be an indicator of dentoskeletal malocclusion in the early stage (ranging from 4 to 12 years old). It indicates that the forehead and mandibular, as part of the craniomaxillofacial complex, interact with each other’s growth and development. To some extent, there may be some regulatory mechanism that enhances the development of the mandible in children with pronounced prominent foreheads. And also inhibits or retards the development of the frontal and maxillary bones during puberty. This ultimately leads to dentoskeletal malocclusion Class III with the profile of a prominent mandible and flat forehead in adulthood. Therefore, the protrusion, morphology, and developmental regularity of forehead should be considered carefully in the clinic. For plastic surgeons, our study can be a reference standard for determining the morphology and protrusion of forehead. And for orthodontists, treatment plans should take forehead protrusion and profile into consideration.

### Frontal sinus

The frontal sinus was noticeable when the forehead was scanned on lateral radiographs. In contrast to other sinus cavities, the frontal sinus was absent at birth [31]. During the fourth and fifth weeks of gestation, the frontal sinus begins to develop at a very slow speed [23]. At as young as 2 years of age, it begins to grow. It becomes radiographically evident at around 6 or 8 years old, which is the first rapid growth stage, and the next period occurs at the onset of puberty, at nearly 20 years of age when the shape and size of the frontal sinus become stable [24]. In our measurements of 405 samples, we found that the frontal sinus depth in adults and adolescents was greater than that in children, indicating that the frontal sinuses may develop significantly during adolescence.

According to previous studies, frontal sinus development was associated with dentoskeletal deformities. The anteroposterior dimension of the frontal sinus decreased in vertical growth patterns [32]. Through exploring the relationship between the frontal sinus area and malocclusion deformities, the study of Said et al. has shown that there was a significant difference in frontal sinus area between Class I and all malocclusion groups [33]. It has been reported that frontal sinus area reduction after 6 months of maxillomandibular advancement counterclockwise rotation for class II anterior open-bite malocclusion [34]. In a study by Murat Tunca [35], the length and height of frontal sinus were found to be higher in dentoskeletal Class III individuals than in dentoskeletal Class I and II individuals. However, no significant difference was found in the frontal sinus width among the three dentoskeletal categories, both in all samples and all age subgroups in our study. Frontal sinus is a three-dimensional structure, the area presented on the lateral radiographs may be erroneous by the location of the head. Therefore, sagittal measurement of width may be more superior on two-dimensional radiographs.

Frontal sinus development can cause substantial variation in the shape of the medial forehead. Marked fullness over the frontal sinuses is more common in men than in women. Noteworthy, the present study showed that dentoskeletal deformities impair the correlation between the frontal sinuses and forehead protrusion, by affecting the size of the frontal sinuses or the shape of the forehead in development. Attention should be paid to the relationship between forehead protrusion and frontal sinus of adult patients, which may be used as a reference indicator for the normality of maxillofacial structures.

### Forehead and aesthetics

It’s worth mentioning that forehead and chin are the first noticed areas, particularly on female concave faces, reflecting their status as the reference standards for esthetic evaluations in our unpublished data. At the same time, aesthetic analysis cannot leave any part of the face behind, especially the forehead. Moreover, our unpublished study found that forehead morphology influences the coordination of the entire face, the greater the prominence of the forehead, the higher the perceived attractiveness of a mildly protruding nose perceived attractiveness, and it was more acceptable for mildly protruding lips with a prominent forehead and flat lips with a flatter forehead, while a normal forehead protrusion accepted a wide range of chin protrusions.

Therefore, the protrusion, morphology, and developmental regularity of forehead should be considered carefully in clinics. The study can provide a theoretical basis for overall esthetic research and clinics in the future. Based on this research, we can systematically study the esthetic paradigm of facial soft tissue, considering individual characteristics and determining the ideal proportions of each face to create “individual beauty”.

### Limitation and future study

One limitation of the study is the sample size. Although our sample size is larger than the number of minimum estimates (*n* = 96, 95% CI, α = 0.05, accuracy level = 10%), a larger number of participants may improve the accuracy of future studies. This study was conducted from the perspective of the craniomaxillofacial developmental complex, whereas the current study only explored the craniofacial sagittal direction, the relationship among more anatomical landmarks can be studied from a multidimensional perspective in the future.

## Conclusions

In conclusion, forehead morphology and protrusion are related to sex, age, race, and dentoskeletal malocclusion, and the frontal bone may correlate with the development of the mandibular, suggesting that forehead morphology and protrusion should be considered in orthodontic clinical practice.

## Data Availability

The data that support the findings of this study are available from the corresponding author upon reasonable request.

## References

[1] Baker RS, Fields HJ, Beck FM, Firestone AR, Rosenstiel SF (2018). Objective assessment of the contribution of dental esthetics and facial attractiveness in men via eye tracking. Am J Orthod Dentofac Orthop.

[2] Dondzilo L, Spring S, MacLeod C (2022). Effect of manipulating facial attractiveness judgements on the experience of intrusive thoughts in high facial appearance concern individuals. Behav Res Ther.

[3] Maisel A, Waldman A, Furlan K (2018). Self-reported patient motivations for seeking cosmetic procedures. JAMA Dermatol.

[4] Toti Ç, Kaςani G, Meto A, Droboniku E, Gurakuqi A, Tanellari O, Hysi D, Fiorillo L (2023). Early Treatment of Class II Division 1 Malocclusions with Prefabricated Myofunctional Appliances: A Case Report. Prosthesis.

[5] Phillips C, Bennett ME, Broder HL (1998). Dentofacial disharmony: psychological status of patients seeking treatment consultation. Angle Orthod.

[6] Deng X, Wang YJ, Deng F, Liu PL, Wu Y (2018). Psychological well-being, dental esthetics, and psychosocial impacts in adolescent orthodontic patients: a prospective longitudinal study. Am J Orthod Dentofac Orthop.

[7] Crimi S, Defila L, Nanni M, Cicciu M, Fiorillo L, Cervino G, Marchetti C, Bianchi A (2020). Three-dimensional evaluation on cortical bone during orthodontic surgical treatment. J Craniofac Surg.

[8] Ackerman JL, Proffit WR, Sarver DM (1999). The emerging soft tissue paradigm in orthodontic diagnosis and treatment planning. Clin Orthod Res.

[9] Ghorbanyjavadpour F, Rakhshan V (2019). Factors associated with the beauty of soft-tissue profile. Am J Orthod Dentofac Orthop.

[10] Seo KH, So DH, Song KT, Choi SK, Kang KH (2021). Effect of lower facial height and anteroposterior lip position on esthetic preference for Korean Silhouette profiles. Korean J Orthod.

[11] Zarif NH, Sabouri SA, Ebrahimi E, Torkan S (2016). Esthetic evaluation of lip position in Silhouette with respect to profile divergence. Am J Orthod Dentofac Orthop.

[12] Garritano FG, Quatela VC (2018). Surgical anatomy of the upper face and forehead. Facial Plast Surg.

[13] Fedok FG (2018). The aesthetics of the upper face: forehead, brow, and upper eyelid. Facial Plast Surg.

[14] Knoll BI, Attkiss KJ, Persing JA (2008). The influence of forehead, brow, and periorbital aesthetics on perceived expression in the youthful face. Plast Reconstr Surg.

[15] Baimati M. Analysis of differences in perception of Oral and maxillofacial aesthetics among different Populations. MA theses. Jilin University. 2022. 10.27162/d.cnki.gjlin.2022.005876.

[16] Huijuan G. The study of the esthetic evaluation in facial soft tissue profile and its sensitivity Indexs of female in northeast China. MA theses. China Medical University. 2020. 10.27652/d.cnki.gzyku.2020.001522.

[17] Salehi P, Oshagh M, Aleyasin ZS, Pakshir HR (2014). The effects of forehead and neck position on esthetics of class I, II and III profiles. Int J Esthet Dent.

[18] Powell N, Humphreys B (1984). Proportions of the aesthetic face.

[19] Wang SX, Zhao JJ, Feng Y, Liu ZX (2014). An analysis of soft tissue profile features of beautiful men and women with polar coordinates. Shanghai Journal of Stomatology.

[20] Yang Y, Cao M, Fu S, Gui L, Xuan S, Yang X, Li Z, Ding Y (2016). Initial analysis of the profile of Xi'an adults with individual Normal occlusion. Chinese Journal of Conservative Dentistry.

[21] Kocandrlova K, Dupej J, Hoffmannova E, Veleminska J (2021). Three-dimensional mixed longitudinal study of facial growth changes and variability of facial form in preschool children using Stereophotogrammetry. Orthod Craniofacial Res.

[22] Brons S, Meulstee JW, Nada RM, Kuijpers M, Bronkhorst EM, Berge SJ, Maal T, Kuijpers-Jagtman AM (2019). Uniform 3D meshes to establish normative facial averages of healthy infants during the first year of life. PLoS One.

[23] Moore K, Ross A (2017). Frontal sinus development and juvenile age estimation. Anat Rec (Hoboken).

[24] Sardi ML, Joosten GG, Pandiani CD, Gould MM, Anzelmo M, Ventrice F (2018). Frontal sinus ontogeny and covariation with bone structures in a modern human population. J Morphol.

[25] Mahmood HT, Shaikh A, Fida M (2016). Association between frontal sinus morphology and cervical vertebral maturation for the assessment of skeletal maturity. Am J Orthod Dentofac Orthop.

[26] Sykes JM, Moore EJ (2002). Esthetic contouring of the forehead and supraorbital rims. Facial Plast Surg Clin North Am.

[27] Holdaway RA (1983). A soft-tissue cephalometric analysis and its use in orthodontic treatment planning. Part I Am J Orthod.

[28] Legan HL, Burstone CJ (1980). Soft tissue cephalometric analysis for Orthognathic surgery. J Oral Surg.

[29] McKinney P, Mossie RD, Zukowski ML (1991). Criteria for the forehead lift. Aesthet Plast Surg.

[30] Goldstein SM, Katowitz JA (2005). The male eyebrow: a topographic anatomic analysis. Ophthalmic Plast Reconstr Surg.

[31] Tabor Z, Karpisz D, Wojnar L, Kowalski P (2009). An automatic recognition of the frontal sinus in X-ray images of skull. IEEE Trans Biomed Eng.

[32] Metin-Gursoy G, Akay G, Balos TB (2021). Frontal sinus: is it a predictor for vertical malocclusions?. Anat Sci Int.

[33] Said OT, Rossouw PE, Fishman LS, Feng C (2017). Relationship between anterior occlusion and frontal sinus size. Angle Orthod.

[34] Prado FB, Rossi AC, Freire AR, Groppo FC, De Moraes M, Caria PH (2012). Pharyngeal airway space and frontal and sphenoid sinus changes after Maxillomandibular advancement with counterclockwise rotation for class II anterior open bite malocclusions. Dentomaxillofac Radiol.

[35] Tunca M, Kaplan V, Kaya Y, Tunca Y (2022). The relationship between frontal sinus dimensions and skeletal malocclusion. Eur Oral Res.

